# A Comparative Analysis of Dexmedetomidine Infusion Rates on Hemodynamic Responses During Laparoscopic Cholecystectomy: An Observational Study

**DOI:** 10.7759/cureus.74538

**Published:** 2024-11-26

**Authors:** Rachna Verma, Amra Nadeem, Narendra S Bafila, Sanni D Gautam, Deepak Kumar, Manoj K Giri

**Affiliations:** 1 Anaesthesiology, Ganesh Shankar Vidyarthi Memorial Medical College, Kanpur, IND; 2 Anaesthesiology, Dr. KNS Memorial Institute of Medical Sciences, Barabanki, IND; 3 Anaesthesiology, Era’s Lucknow Medical College and Hospital, Lucknow, IND; 4 Anaesthesia and Critical Care, Dr. Ram Manohar Lohia Institute of Medical Sciences, Lucknow, IND

**Keywords:** anesthetic adjuvant, dexmedetomidine, dose comparison, hemodynamic stability, laparoscopic cholecystectomy

## Abstract

Background

Although laparoscopic surgery is becoming more common, its anesthetic management poses challenges due to hemodynamic fluctuations. Dexmedetomidine has shown promise in mitigating these responses. In this study, we compared the effects of three different infusion doses of dexmedetomidine (0.2 µg/kg/hour, 0.4 µg/kg/hour, and 0.6 µg/kg/hour) on hemodynamic responses at the time of laparoscopic cholecystectomy.

Methodology

An observational study was conducted among 90 adult patients undergoing laparoscopic cholecystectomy. Patients were divided into three groups, each receiving one of the three dexmedetomidine doses. Hemodynamic parameters (systolic and diastolic blood pressures, mean arterial pressure, etc.) were monitored at various time points throughout the procedure.

Results

All three dexmedetomidine doses effectively attenuated hemodynamic responses. The 0.4 µg/kg/hour dose demonstrated the most stable blood pressure control, with minimal fluctuations from baseline. The 0.6 µg/kg/hour dose consistently resulted in lower mean blood pressure values. Heart rate was also more stable in the 0.4 µg/kg/hour group, while the 0.6 µg/kg/hour group consistently maintained lower heart rates.

Conclusions

Dexmedetomidine is a valuable anesthetic adjuvant for laparoscopic cholecystectomy, with the 0.4 µg/kg/hour dose offering a favorable balance between hemodynamic stability and the potential risk of hypotension and bradycardia.

## Introduction

Laparoscopic surgery has significantly expanded in both scope and volume since its inception in diagnostic procedures in the 1970s and the groundbreaking cholecystectomy in the 1980s [1]. These minimally invasive procedures offer several advantages, including reduced morbidity, mortality, and length of hospital stay, as well as decreased postoperative pain, lower healthcare costs, and cosmetically appealing scars [1-3].

However, the anesthetic management of laparoscopic surgery imposes challenges due to the pathophysiological changes associated with pneumoperitoneum, primarily increased systemic as well as pulmonary vascular resistance, elevated heart rate, and decreased cardiac output [4]. The patient’s position during the procedure can further exacerbate these hemodynamic alterations [5]. Additionally, carbon dioxide absorption during pneumoperitoneum can lead to hypercapnia, increasing anesthetic complexity. Moreover, the stimulation of the sympathetic nervous system during serious conditions, viz. laryngoscopy, intubation, and extubation, can induce significant hemodynamic fluctuations [6]. Collectively, these factors necessitate careful anesthetic management.

Various agents, including isoflurane, propofol, β-blockers, antihypertensives, and α2-adrenergic agonists such as clonidine, have been explored to mitigate the hemodynamic responses associated with laparoscopic surgery [1]. These agents may decrease anesthetic and analgesic requirements, improve perioperative hemodynamic stability, and attenuate neuroendocrine stress response linked with major surgery.

Dexmedetomidine, approved by the Food and Drug Administration (FDA) in 1999, is a sedative that binds strongly to α2-adrenergic receptors [7]. Compared to clonidine, it has minimal effects on α1 receptors. It stipulates anxiolysis as well as sedation without causing significant respiratory depression. By decreasing central nervous system (CNS) sympathetic outflow in a dose-dependent manner, it offers opioid-sparing analgesia and has demonstrated organ-protective effects, including cardioprotection, neuroprotection, and renoprotection [8]. When administered intravenously, dexmedetomidine helps reduce the body’s stress response during procedures such as laryngoscopy, intubation, and/or surgery [9,10]. This is because it calms the nervous system and minimizes heart rate and blood pressure [11]. While it can cause some drowsiness, it typically does not significantly affect breathing. Originally used for intensive care unit (ICU) sedation, it is now employed as an anesthetic adjuvant due to its significant benefits. Moreover, it has been shown to reduce the requirement for induction agents and opioids perioperatively [1,12].

To better understand how dexmedetomidine affects blood pressure and heart rate during laparoscopic cholecystectomy, several studies have investigated different dosing strategies and administration methods [13-17]. However, the findings of these studies have been conflicting. Here, we hypothesized that the administration of dexmedetomidine at an infusion rate of 0.4 µg/kg/hour would result in the most stable hemodynamic profile during laparoscopic cholecystectomy compared to infusion rates of 0.2 µg/kg/hour and 0.6 µg/kg/hour. We aimed to compare the effect of three different doses (0.2 µg/kg/hour, 0.4 µg/kg/hour, and 0.6 µg/kg/hour) of dexmedetomidine on hemodynamics in patients undergoing laparoscopic cholecystectomy under general anesthesia. Objectives of the study were (i) to monitor the ameliorating effect of three different doses of dexmedetomidine on hemodynamic changes (heart rate, blood pressure, and mean arterial pressure (MAP)) in patients undergoing laparoscopic cholecystectomy under general anesthesia; (ii) to determine the most appropriate doses of dexmedetomidine for the above; and (iii) to assess the side effects, if any, due to study drugs.

## Materials and methods

Study design and setting

**Figure 1:**
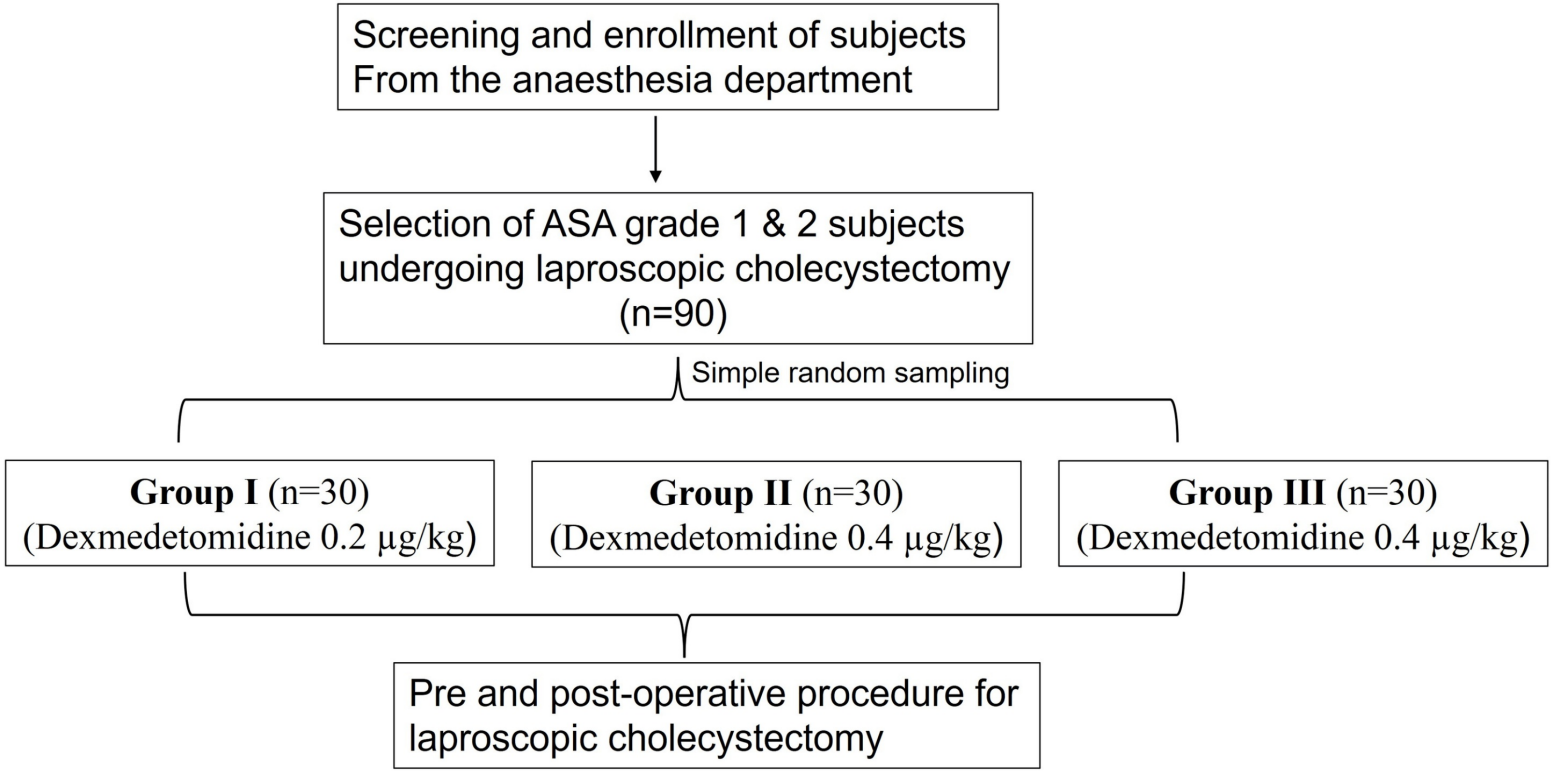
Study flowchart. ASA: American Society of Anesthesiologists

In this prospective observational study, 90 American Society of Anesthesiologists Grade I and II patients selected for laparoscopic cholecystectomy under anesthesia were enrolled. Further, patients were divided into three groups (0.2 µg/kg/hour, 0.4 µg/kg/hour, and 0.6 µg/kg/hour) of 30 patients each by using a simple randomization method. All patients were informed about the procedure and the study drug and were given the opportunity to ask questions (Figure 1). This study was approved by the Institutional Ethics Committee (approval number: ELMC/R.Cell/EC/2017/34) and conducted in accordance with the approved protocol. Additionally, written informed consent was obtained from all patients before their inclusion in the study.

Sample size calculation

The sample size was calculated using the following formula: n = (α_1_^2^ + α_2_^2^)(Z_α_ + Z_β_)^2^/d^2^, where α1 = 13.132, α2 = 7.59 SDs of both groups, d = mean (a1, a2), the difference considered to be medically significant, type I error α = 5%, and type II error β = 10% for detecting results with 90% power of study. Data loss was considered at 10%. The sample size was calculated to be 30 in each group. A total of 90 patients were enrolled in the study, who were further randomly allocated into three groups of 30 patients each.

Inclusion and exclusion criteria

Adult patients aged 18 to 65 years with ASA Grade I or II who were scheduled for elective laparoscopic cholecystectomy under anesthesia were enrolled in the study. Patients with any of the following comorbidities were excluded: chronic obstructive pulmonary disease; morbid obesity; hepatic, endocrine, or cardiac dysfunction; hypertension; and diabetes mellitus. Patients who were unable to be intubated on the first attempt, anticipated to have difficulty with intubation, or expected to undergo a procedure lasting longer than 90 minutes were also excluded.

Surgical procedure

Initially, a pre-anesthetic evaluation was conducted, and informed consent for anesthesia and the study was obtained. Patients were fasted overnight, and intravenous cannulation with an 18-G cannula was performed in the preoperative area on the morning of the surgery. The infusion was prepared for each group by a separate person. After shifting the patient to the operation theater, the monitors were attached and baseline vital parameters were recorded. Study drugs were administered as follows: Group I: injection dexmedetomidine 0.2 µg/kg intravenous over 10 minutes; Group II: injection dexmedetomidine 0.4 µg/kg intravenous over 10 minutes; Group III: injection dexmedetomidine 0.6 µg/kg intravenous over 10 minutes just before induction and then followed by maintenance infusion at the rate of 0.2 µg/kg/hour (Group I), 0.4 µg/kg/hour (Group II), and 0.6 µg/kg/hour (Group III), respectively, till the release of pneumoperitoneum.

Patients were pre-oxygenated (100% oxygen) for three minutes and premedicated with fentanyl (2 µg/kg). Induction was performed with propofol (2 mg/kg), and endotracheal intubation was facilitated with vecuronium (0.1 mg/kg). Further, induction was done with injection propofol (2 mg/kg). Subsequently, endotracheal intubation was performed with an appropriately sized endotracheal tube following the administration of injection vecuronium 0.1 mg/kg intravenously. After securing the airway, anesthesia was maintained in all three groups by nitrous oxide + oxygen (50:50) with isoflurane as the volatile agent. Incremental doses of injection vecuronium (0.02 mg/kg) were supplemented to maintain the intraoperative relaxation. At the end of the surgery, neuromuscular blockade was reversed with injection neostigmine (50 µg/kg) and injection glycopyrrolate (10 µg/kg) once the spontaneous respiratory efforts were back. The heart rate, systolic blood pressure (SBP), diastolic blood pressure (DBP), and MAP were noted at the following intervals: preoperative (just before bolus administration of dexmedetomidine) (T0); immediately after bolus administration of dexmedetomidine (T1); one minute after induction of anesthesia (T2); one minute after endotracheal intubation (T3); immediately after the creation of pneumoperitoneum (T4); every 15 minutes thereafter until the release of pneumoperitoneum (T5 to T10); after the release of pneumoperitoneum (coinciding with the end of drug infusion) (T11), and in the postoperative period (T12).

Data analysis

Demographics and clinical parameters were recorded on individual case record forms. These data were then transferred to a Microsoft Excel spreadsheet (Microsoft Corp., Redmond, WA, USA) for analysis. Further, data were presented as number (n), percentage (%), mean, and standard deviation. SPSS software (IBM Corp., Armonk, NY, USA) was utilized to perform data analysis. The chi-square test was used to compare categorical variables such as gender distribution and ASA grade between the groups. For continuous variables such as age, the Student’s t-test was used to assess any statistically significant differences between the groups at baseline. Mean differences in SBP, DBP, MAP, and heart rate were assessed along with the change in SBP, DBP, MAP, and heart rate (compared to baseline) between the groups at each time point individually. Paired t-tests were used to compare the mean difference among groups at each time point. P-values <0.05 were considered statistically significant.

## Results

Baseline characteristics

Baseline characteristics are presented in Table 1. There was no significant difference among the mean age of participants: Group I (37.40 ± 8.21 years), Group II (35.67 ± 9.31 years), and Group III (37.37 ± 10.55 years) (p = 0.65). Group I had nine (70.0%) males and 21 (30.0%) females, Group II had 16 (53.3%) males and 14 (46.7%) females, and Group III had 17 (56.7%) males and 13 (43.3%) females. The ASA grade distribution was also analyzed, with Group I having 46.7% (n = 14) Grade 1 patients and Group II having 53.3% (n = 16) Grade 2 patients. The preoperative measurements of SBP, DBP, MAP, and heart rate measurements were also recorded (Table 1). Baseline hemodynamic variables (SBP, DBP, MAP, and heart rate) of patients of the three groups were found to be comparable.

**Table 1:** Demographic and clinical variables of the study groups. The chi-square test and Student’s t-test were used to calculate the p-value. ASA: American Society of Anesthesiologists; SBP: systolic blood pressure; DBP: diastolic blood pressure; MAP: mean arterial pressure

Variables		Group I (n = 30)	Group II (n = 30)	Group III (n = 30)	P-value
Age (years), mean ± SD		37.40 ± 8.21	35.67 ± 9.31	37.37 ± 10.55	0.675
Gender	Male	9 (70.0)	16 (53.3)	17 (56.7)	0.10
	Female	21 (30.0)	14 (46.7)	13 (43.3)	
ASA grade	Grade1	14 (46.7)	16 (53.3)	15 (50.0)	0.875
	Grade 2	16 (53.3)	14 (46.7)	15 (50.0)	
Time interval (preoperative), mean ± SD	SBP	132.13 ± 3.95	131.67 ± 4.74	130.07 ± 7.09	0.307
	DBP	84.77 ± 5.69	84.67 ± 6.17	83.20 ± 6.30	0.536
	MAP	100.56 ± 5.08	100.32 ± 5.58	98.78 ± 5.80	0.402
	Heart rate	87.27 ± 6.89	87.10 ± 9.16	87.80 ± 6.74	0.934

Mean difference in systolic blood pressure based on time interval

Table 2 shows the mean difference in SBP among study groups at T0 to T12. No significant differences were observed at T0, but from T1 to T4, Group III consistently had the highest SBP, followed by Group I and Group II. At T5, a negative difference was noted between Group I and Group II, but no significant changes were seen between Group I and Group III. However, there was a significant difference between Group II and Group III. From T6 to T11, no significant changes were observed, and at T12, significant differences were found between Group I and Group II and Group I and Group III, with no significant changes between Group II and Group III.

**Table 2:** Mean differences in systolic blood pressure between all study groups based on time intervals. The paired t-test was used to calculate the mean differences between the study groups. P-values <0.05 were considered statistically significant. T0: preoperative; T1: after bolus drug administration; T2: one minute after induction; T3: one minute after intubation; T4: after pneumoperitoneum; T5: 15 minutes; T6: 30 minutes; T7: 45 minutes; T8: 60 minutes; T9: 75 minutes; T10: 90 minutes; T11: release of pneumoperitoneum (coinciding with the end of drug infusion); T12: postoperative period; T: time interval; r: mean differences

Time interval	Group I vs. Group II		Group I vs. Group III		Group II vs. Group III	
	r mean	P-value	r mean	P-value	r mean	P-value
T0	0.47	0.941	2.07	0.308	1.60	0.491
T1	11.47	<0.0001	22.13	<0.0001	10.67	<0.0001
T2	15.70	<0.0001	29.27	<0.0001	13.57	<0.0001
T3	16.10	<0.0001	19.90	<0.0001	3.80	0.010
T4	11.70	<0.0001	15.93	<0.0001	4.23	<0.0001
T5	-4.67	<0.0001	-0.03	1.000	4.63	<0.0001
T6	-1.73	0.563	1.00	0.825	2.73	0.244
T7	-1.40	0.763	1.30	0.792	2.70	0.369
T8	1.60	0.675	3.90	0.103	2.30	0.446
T9	1.73	0.753	2.00	0.686	0.27	0.993
T10	1.93	0.692	2.33	0.586	0.40	0.984
T11	0.10	0.999	0.77	0.940	0.67	0.955
T12	9.83	<0.0001	13.37	<0.0001	3.53	0.374

Mean difference in diastolic blood pressure among all the study groups

The study found no significant differences between Group I and Group II, Group I and Group III, and Group II and Group III at the initial time (Table 3). However, significant differences were observed at T1 and T2, with Group I and Group II showing significant differences. From T4 to T10, significant differences were consistently observed between Group I and Group II, with notable differences at T6 and T7. Group II and Group III also showed consistent significance across these intervals. Significant differences persisted between Group I and Group III and between Group II and Group III.

**Table 3:** Mean differences in diastolic blood pressure between all study groups based on time intervals. The paired t-test was used to calculate the mean differences between the study groups. P-values <0.05 were considered statistically significant. T0: preoperative; T1: after bolus drug administration; T2: one minute after induction; T3: one minute after intubation; T4: after pneumoperitoneum; T5: 15 minutes; T6: 30 minutes; T7: 45 minutes; T8: 60 minutes; T9: 75 minutes; T10: 90 minutes; T11: release of pneumoperitoneum (coinciding with the end of drug infusion); T12: postoperative period; T: time interval; r: mean differences

Time interval	Group I vs. Group II		Group I vs. Group III		Group II vs. Group III	
	Mean	P-value	Mean	P-value	Mean	P-value
T0	0.10	0.998	1.57	0.578	1.47	0.618
T1	-4.23	0.043*	0.03	1.000	4.27	0.041*
T2	-9.47	<0.0001*	-0.60	0.936	8.87	<0.0001*
T3	-2.47	0.512	0.30	0.990	2.77	0.431
T4	-6.37	<0.0001*	-3.53	0.048	2.83	0.137
T5	-4.70	<0.0001*	0.30	0.958	5.00	<0.0001*
T6	-8.33	<0.0001*	-1.43	0.651	6.90	<0.0001*
T7	-7.40	<0.0001*	-0.13	0.996	7.27	<0.0001*
T8	-7.23	<0.0001*	0.03	1.000	7.27	<0.0001*
T9	-6.23	0.001*	0.53	0.944	6.77	<0.0001*
T10	-5.67	0.008*	1.03	0.841	6.70	0.001*
T11	2.57	0.572	9.27	0.001	6.70	0.027*
T12	2.77	0.464	10.80	<0.001	8.03	0.003*

Mean difference in mean arterial pressure based on time interval in the study groups

At T0, the mean differences were not statistically significant (Table 4). However, at T1, significant changes were found between Group I and Group III and Group II and Group III, while Group I and Group II did not show a significant difference. The pattern continued at T2, where Group I vs. Group III showed high significance, while Group I vs. Group II did not. At T3, significant changes were observed between Group I and Group III, and a marginal significance between Group I and Group II. Subsequent intervals (T4 to T12) showed varying levels of significance, with notable differences at T4 between Group I and Group III and Group II and Group III. At T5, significant differences were observed between Group I and Group II and Group II and Group III. This trend continued through T6 to T12, with several significant differences between the groups, indicating a dynamic relationship over time.

**Table 4:** Mean differences in mean arterial pressure between all study groups based on time intervals. The paired t-test was used to calculate the mean differences between the study groups. P-values <0.05 were considered statistically significant. T0: preoperative; T1: after bolus drug administration; T2: one minute after induction; T3: one minute after intubation; T4: after pneumoperitoneum; T5: 15 minutes; T6: 30 minutes; T7: 45 minutes; T8: 60 minutes; T9: 75 minutes; T10: 90 minutes; T11: release of pneumoperitoneum (coinciding with the end of drug infusion); T12: postoperative period; T: time interval; r: mean differences

Time interval	Group I vs. Group II		Group I vs. Group III		Group II vs. Group III	
	Mean	P-value	Mean	P-value	Mean	P-value
T0	0.23	0.985	1.77	0.428	1.54	0.526
T1	1.00	0.765	7.40	<0.0001*	6.40	<0.0001*
T2	-1.08	0.737	9.36	<0.0001*	10.43	<0.0001*
T3	3.72	0.046	6.83	<0.0001*	3.11	0.113
T4	-0.34	0.940	2.96	0.013*	3.30	0.005*
T5	-4.69	<0.0001*	0.19	0.981	4.88	<0.0001*
T6	-6.13	<0.0001*	-0.62	0.915	5.51	0.002*
T7	-5.40	0.004*	0.34	0.976	5.74	0.002*
T8	-4.29	0.009*	1.32	0.621	5.61	<0.0001*
T9	-3.58	0.076	1.02	0.804	4.60	0.016*
T10	-3.13	0.183	1.47	0.684	4.60	0.028*
T11	1.86	0.707	6.55	0.017*	4.69	0.117
T12	5.12	0.072	11.66	<0.0001*	6.53	0.015

Mean difference in heart rate among all the study groups

There were no significant changes in heart rate among groups at different time intervals (Table 5). At T0, there were no significant differences between Group I and Group II, Group I and Group III, and Group II and Group III. However, from T1 onward, significant differences were seen, with Group I vs. Group II showing a Δ mean of 5.73 (p = 0.008) and Group I vs. Group III showing a Δ mean of 10.80 (p < 0.0001). The trend continued across subsequent intervals, with Group I vs. Group III consistently showing the highest mean differences. Group I vs. Group II also showed significant differences, particularly at T6 and T7. The differences between Group II and Group III remained significant throughout, with notable mean differences at T12.

**Table 5:** Mean differences in heart rate between all study groups based on time intervals. The paired t-test was used to calculate the mean differences between the study groups. P-values <0.05 were considered statistically significant. T0: preoperative; T1: after bolus drug administration; T2: one minute after induction; T3: one minute after intubation; T4: after pneumoperitoneum; T5: 15 minutes; T6: 30 minutes; T7: 45 minutes; T8: 60 minutes; T9: 75 minutes; T10: 90 minutes; T11: release of pneumoperitoneum (coinciding with the end of drug infusion); T12: postoperative period; T: time interval; r: mean differences

Time interval	Group I vs. Group II		Group I vs. Group III		Group II vs. Group III	
	Mean	P-value	Mean	P-value	Mean	P-value
T0	0.23	0.992	-0.37	0.981	-0.60	0.951
T1	5.73	0.008*	10.80	<0.0001*	5.07	0.021*
T2	11.40	<0.0001*	16.13	<0.0001*	4.73	0.030*
T3	7.17	0.001*	13.93	<0.0001*	6.77	0.001*
T4	11.67	<0.0001*	17.93	<0.0001*	6.27	0.002*
T5	15.20	<0.0001*	21.47	<0.0001*	6.27	0.002*
T6	18.70	<0.0001*	24.03	<0.0001*	5.33	0.008*
T7	22.63	<0.0001*	27.53	<0.0001*	4.90	0.019*
T8	25.87	<0.0001*	30.57	<0.0001*	4.70	0.027*
T9	29.10	<0.0001*	33.67	<0.0001*	4.57	0.030*
T10	32.23	<0.0001*	37.07	<0.0001*	4.83	0.021*
T11	25.13	<0.0001*	29.60	<0.0001*	4.47	0.029*
T12	7.60	<0.0001*	20.63	<0.0001*	13.03	<0.0001*

## Discussion

This study aimed to evaluate the comparative effects of different interventions (dexmedetomidine 0.2 µg/kg/hour, 0.4 µg/kg/hour, 0.6 µg/kg/hour) on SBP, DBP, MAP, and heart rate across three groups over various time intervals. Selection of these three dosages was done keeping in view the safety. Although studies [18-21] have reported the use of doses up to 1 µg/kg, considering patient safety as the paramount factor for decision-making, we decided on a maximum dose of 0.6 µg/kg.

The baseline characteristics of the three groups were comparable, ensuring a well-matched study population. The age of participants in all groups was similar, with no significant differences observed (p = 0.675). Pathak et al. [22] similar to our study reported the mean age of patients between 30 and 50 years. In general, most studies had excluded patients above 60 years of age. In the present study as well, none of the patients were above 60 years of age. The gender distribution was balanced across the groups, with a slight male predominance in Group I and Group III.

The ASA grade distribution was comparable, indicating a similar level of surgical risk among participants. Preoperative hemodynamic parameters (SBP, DBP, MAP, and heart rate) were within normal limits and did not differ significantly between the groups, suggesting a balanced baseline condition, similar to other studies [21-23]. Gautam et al. [21] concluded that dexmedetomidine effectively attenuates fluctuations in the hemodynamics of hypertensive patients, and the attenuation of these hemodynamic variations is not affected by the maintenance dose of 0.5 or 0.7 µg/kg/hour. In another study [5], investigators examined the effects of dexmedetomidine (normal saline group, 0.2 µg/kg/hour and 0.4 µg/kg/hour) infusion on the hemodynamic stress response, sedation, and postoperative analgesia requirements in patients undergoing laparoscopic cholecystectomy. They found that both doses (0.2 µg/kg/hour and 0.4 µg/kg/hour) of dexmedetomidine were effective in reducing the stress response to procedures such as intubation and creating air in the abdomen during surgery. However, the higher dose appeared to be more effective. Additionally, patients who received dexmedetomidine required less pain medication after surgery. The medication was well-tolerated, with no significant side effects reported.

The analysis of DBP revealed a similar trend, with significant differences emerging from T1 onwards. Group I and Group II showed significant differences at T1 and T2, with Group II generally exhibiting lower DBP values compared to Group I. This trend may be attributed to the vasodilatory effects of the interventions used in Group II, which is consistent with previous studies that have reported similar outcomes [5,21]. The persistent significant differences between Group II and Group III throughout the study period further underscore the impact of these interventions on DBP. Interestingly, at T11 and T12, Group I and Group III displayed the most significant differences, suggesting that the effects of the interventions in Group III were more prolonged and pronounced, potentially leading to a sustained increase in DBP.

The MAP is a crucial indicator of organ perfusion. It represents the average pressure in the arteries during a cardiac cycle and is determined by a balance between SBP and DBP. Adequate MAP is essential for optimal blood flow to vital organs. Higher dexmedetomidine doses (0.4 and 0.6 µg) improved hemodynamic stability compared to lower doses (0.2 µg). Our study found that 0.4 µg provided optimal stability, balancing minimal fluctuations with adequate blood pressure control. Unlike previous studies, we did not observe MAP exceeding baseline values, indicating effective pressor response suppression. Our comparison of three incremental doses uniquely identified 0.4 µg as the most stable, aligning with findings that 0.4 µg is superior to 0.2 µg, while 0.6 µg resulted in the lowest MAP values. These findings align with previous research [5,23], which highlighted the superiority of 0.4 µg over 0.2 µg in attenuating hemodynamic responses. Our data showed significant differences particularly between Group I and Group III and Group II and Group III at multiple time points. The most notable differences were observed between Group I and Group III at T1, T2, and T3, where Group III consistently had higher MAP values. These findings are in line with literature that suggests certain pharmacological agents can have a prolonged effect on vascular tone, thereby influencing MAP more significantly [16,17,21]. The significant differences between Groups II and III at T5 and T12 further emphasize the differing pharmacodynamics of the interventions used in these groups. The lack of significant differences at certain intervals, such as T6 and T7, might indicate a period of stabilization in MAP, potentially due to the equilibration of drug effects or compensatory physiological mechanisms. The variation in MAP across the intervals also points to the potential impact of anesthetic management, fluid balance, and postoperative care, which are known to influence MAP.

Regarding heart rate, Masoori et al. [24], while comparing 0.3 µg and 0.6 µg values, did not find a significant difference between the two groups up to two minutes after intubation. However, investigators found the mean heart rate to be significantly lower in the 0.6 µg group compared to the 0.3 µg group at 3-7 minutes and 20-30 minutes post-intubation. Despite this, both groups exhibited mean heart rate values lower than baseline. Therefore, in terms of stability, the 0.3 µg group was more stable than the 0.6 µg group, which showed higher fluctuations from baseline values. Nevertheless, Masoori et al. [24] considered 0.6 µg to be superior to 0.2 µg. Acharya et al. [11], while comparing 0.3 and 0.6 µg groups, found mean values in the 0.3 µg group to be significantly above baseline values at one minute after intubation, while the 0.6 µg group did not experience such a surge and instead had mean values lower than baseline at this interval. Thus, in terms of controlling the post-intubation surge, their study also found the 0.6 µg group to be superior to the 0.3 µg group. In our study, neither the 0.4 µg nor the 0.6 µg group exhibited a heart rate surge following intubation, suggesting that the 0.4 µg group could be considered more stable. Regarding the superiority of 0.4 µg over 0.2 µg, a few studies, including ours, have found 0.4 µg to be better than 0.2 µg [5,23].

The findings of this study have important clinical implications, particularly in the selection of anesthetic agents or surgical techniques that optimize hemodynamic stability. Our data suggest that while 0.4 µg dexmedetomidine provides a good option with balanced hemodynamics to manage hemodynamic response during laparoscopic cholecystectomy, 0.6 µg provides a better scope for incidental changes in hemodynamics. However, caution is needed while using the 0.6 µg dose and evaluating its safety limits for complications such as bradycardia and hypotension. The differential effects observed across the groups highlight the need for personalized approaches to patient management, especially in individuals with pre-existing cardiovascular conditions.

The limitations of the study include a small sample size and a single-center design; hence, the findings may not be generalizable to a larger population. Additionally, the study did not include a placebo group, which could have provided a stronger control for comparison. The study focused on short-term hemodynamic effects, and long-term outcomes, such as cognitive function and quality of recovery, were not assessed. Further randomized control trials with larger sample sizes and a placebo control are needed to confirm the long-term benefits of dexmedetomidine in laparoscopic surgery.

## Conclusions

The findings of this study suggest that while 0.4 µg dexmedetomidine provides a good option with balanced hemodynamics for the management of hemodynamic response during laparoscopic cholecystectomy, 0.6 µg provides a better scope for incidental changes in hemodynamics. However, one must take caution while using the 0.6 mcg dose and evaluate its safety limits for complications such as bradycardia and hypotension.
